# PMS2-associated Lynch syndrome: Past, present and future

**DOI:** 10.3389/fonc.2023.1127329

**Published:** 2023-02-21

**Authors:** Katarina D. Andini, Maartje Nielsen, Manon Suerink, Noah C. Helderman, Jan Jacob Koornstra, Aysel Ahadova, Matthias Kloor, Marian J.E. Mourits, Klaas Kok, Rolf H. Sijmons, Sanne W. Bajwa–ten Broeke

**Affiliations:** ^1^ Department of Genetics, University Medical Center Groningen, University of Groningen, Groningen, Netherlands; ^2^ Department of Clinical Genetics, Leiden University Medical Center, Leiden, Netherlands; ^3^ Department of Gastroenterology and Hepatology, University Medical Center Groningen, University of Groningen, Groningen, Netherlands; ^4^ Department of Applied Tumour Biology, Institute of Pathology, Heidelberg University Hospital, and Clinical Cooperation Unit Applied Tumor Biology, German Cancer Research Center, Heidelberg, Germany; ^5^ Department of Gynaecology, University Medical Center Groningen, University of Groningen, Groningen, Netherlands

**Keywords:** Lynch syndrome (hereditary nonpolyposis colorectal cancer), mismatch repair (MMR), PMS2 gene, colorectal cancer, endometrial cancer, carcinonogenesis

## Abstract

Carriers of any pathogenic variant in one of the MMR genes (*path_MMR* carriers) were traditionally thought to be at comparable risk of developing a range of different malignancies, foremost colorectal cancer (CRC) and endometrial cancer. However, it is now widely accepted that their cancer risk and cancer spectrum range notably depending on which MMR gene is affected. Moreover, there is increasing evidence that the MMR gene affected also influences the molecular pathogenesis of Lynch syndrome CRC. Although substantial progress has been made over the past decade in understanding these differences, many questions remain unanswered, especially pertaining to *path*_*PMS2* carriers. Recent findings show that, while the cancer risk is relatively low, PMS2-deficient CRCs tend to show more aggressive behaviour and have a worse prognosis than other MMR-deficient CRCs. This, together with lower intratumoral immune infiltration, suggests that PMS2-deficient CRCs might have more in common biologically with sporadic MMR-proficient CRCs than with other MMR-deficient CRCs. These findings could have important consequences for surveillance, chemoprevention and therapeutic strategies (e.g. vaccines). In this review we discuss the current knowledge, current (clinical) challenges and knowledge gaps that should be targeted by future studies.

## Introduction

It has long been thought that germline pathogenic *PMS2* variant carriers (*path_PMS2* carriers) represent only a small minority of Lynch syndrome (LS) patients. However, more recent investigations have revealed that the population frequency of *path_PMS2* carriers is actually the highest among the four mismatch repair (MMR) genes (1 in 714) (1). Germline *path_PMS2* carriers have a much lower risk of developing cancer compared to other *path_MMR* carriers, although the cancer risk seems to vary widely among affected individuals from the same family (2–4). Stratifying LS patients by MMR gene is therefore of vital importance for research and clinical purposes. Indeed, accumulating evidence suggests that PMS2-deficient (dPMS2) tumours show distinct biological behaviour that differs from other MMR-deficient (dMMR) cancers. At the moment, it is still unknown whether the preventive measures now being investigated, such as vaccination or aspirin chemoprevention, would benefit *path_PMS2* carriers in the same way as other *path_MMR* carriers. Given these clinical and research questions, we set out to review the literature on these topics and challenges, discuss clinical scenarios that highlight their importance and identify knowledge gaps to be addressed by future studies.

## Past

### Identification of path_PMS2 carriers

The clinical involvement of *path_PMS2* variants in LS was first described in 1994. However, clinical testing of the gene did not become available until 2009 because the PMS2 gene is notoriously difficult to analyse due to the existence of multiple pseudogenes (5–9). *PMS2* is located on the short arm of chromosome 7 and spans 15 exons. Multiple regions with over 90% homology have been identified, all on chromosome 7, and these pseudogene regions make interpretation of sequencing results of the *PMS2* gene challenging. A variety of strategies, including designing long-range amplicons (9) and RNA analysis (7), have helped overcome this problem and led to improved variant detection and increased identification of *path_PMS2* carriers. Another explanation for the reported underestimation of *PMS2*-LS prevalence lies in selection of families for genetic testing using family history or age of diagnosis (Bethesda and Amsterdam criteria) (10). Previous work has shown that *path_PMS2* variants are predominantly found in families that do not fulfil these criteria (2, 4, 11, 12).

The traditional approach to identifying LS using clinical selection criteria is of limited use in the identification of *path_PMS2* carriers, making it difficult to determine their prevalence. Answers can be sought in population-based colorectal cancer (CRC) cohorts. Studies using immunohistochemical staining (IHC) in CRCs from population-based cohorts have shown that isolated PMS2 loss of expression, indicative of *path_PMS2* variants, is present in 0.5–1.5% of unselected CRCs (2, 12). The fraction of isolated PMS2 loss in MSI CRCs varies between 1–8% (13–15). More than half of such tumours have been shown to be caused by a germline *path_PMS2* variant (16, 17). Another possibility is the presence of double somatic hits, reported to be the cause in 13-17% of isolated dPMS2-CRC (17). Of note, this fraction is lower than for tumours with MLH1/PMS2 or MSH2/MSH6 loss. In other words, isolated PMS2 loss is highly indicative of a germline *path_PMS2* variant. One study of population-based CRCs found a higher percentage of isolated PMS2 than of MSH2 loss of expression (12% versus 11%) in tumours with negative MMR staining (12). Moreover, recent studies have also shown that *PMS2* and *MSH6* variants are much more prevalent in unselected (population-based) cohorts than in those selected by traditional family history criteria. Estimates of (Western) population carrier frequency based on statistical approaches are 1 in 714 for *PMS2* and 1 in 758 for *MSH6*, whereas the prevalences are 1 in 1946 for *MLH1* and 1 in 2841 for *MSH2* (1). Secondly, an unselected study involving the entire Icelandic population found an incidence of 1 in 226 for *PMS2* and *MSH6* variants combined (18). Another finding that may indicate that the carrier frequency of *PMS2* variants is higher than that of *MLH1* and *MSH2* variants involves the fact that biallelic *path_PMS2* variants comprise more than half of the homozygous or compound heterozygous variants in patients reported with the rare early-onset autosomal recessive disorder Constitutional MMR Deficiency (CMMR-D) (31/57) (19). However, this may also result from the lower penetrance of *path_PMS2* variants, which makes them difficult to detect by clinical selection criteria, as will be discussed in the next section. In such a situation, fetuses with biallelic *path_MLH1* or *path_MSH2* variants might be less viable than fetuses with biallelic *path_PMS2* variants, leading to overrepresentation of biallelic *path_PMS2* carriers amongst CMMR-D cases. Of note, the possible occurrence of a child with CMMR-D in *path_PMS2* families, especially consanguineous ones, is an argument for the importance of detecting *path_PMS2* carriers despite the relatively low penetrance.

The most significant improvement in the detection of *path_MMR* carriers is most likely introduction of universal IHC staining of the MMR protein. While the specific screening strategy differs by country, ranging from true universal screening of all CRCs to age-dependent IHC, this approach has proven to be cost-effective and has the added benefit that no additional selection criteria are needed (20–22).

### Cancer risks

The first large cohort of *path_PMS2* carriers (55 index patients and 55 relatives) was reported by Senter et al. in 2008 (2). They reported a cumulative risk for CRC at age 70 years of 20% (95% confidence interval (CI): 11–34%) for male *path_PMS2* carriers and 15% (95% CI: 8–26%) for female *path_PMS2* carriers. The cumulative risk at age 70 for endometrial cancer (EC) was found to be 15% (23). These risks are substantially lower than those previously reported for *path_MLH1*, *path_MSH2* and *path_MSH6* carriers, which range from 25–75% up to age 70 years for CRC and 30–35% for EC.

In 2015, we analysed 98 PMS2 families and found similar cancer risks to those previously reported, i.e. risks of 11–19% for CRC and 12% for EC up to age 70 years (**Table 1**) (4), further supporting that PMS2*-*LS patients face significantly lower risks than other LS patients. This study was underpowered for analyses of less frequent LS-associated cancers. In a second, larger study by our group, consisting of 284 families and providing enough power to also estimate extra-colonic and extra-EC risks, we found increased risk only for CRC and EC (3). The cumulative risks for CRC and EC could be estimated up to age 80 years and were 13–14% and 14%, respectively. Statistically, the cumulative risks for ovarian, gastric, hepatobiliary, bladder, renal, brain, breast, prostate, or small bowel cancer did not significantly deviate from risks in the general population (3). Cancer risks for *path_PMS2* carriers compared to the general population and other *path_MMR* carriers are given in **Table 1**.

**Table 1:** Overview of reported cancer risks.

	General population (lifetime)	Lynch syndrome Barrow et al (up to age 70)*	PMS2-associated Lynch syndrome					
			Senter et al 2008 (95% CI)	Ten Broeke et al 2015 (95% CI)	Ten Broeke et al 2018		Moller et al	
					Up to age 70 (95% CI)	Up to age 80 (95% CI)	IMRC (retrospective)	PLSD (prospective)
**Colorectal**	~4–6%	25–75%	♂: 20% (11–34%)	♂: 19% (6–30%)	♂: 6% (3–13%)	♂: 13% (8–22%)	♂: 7% (6–8%) ♀: 6 (5–6%)	♂:11% (3–37) ♀: 8 (2–29%)
			♀: 15% (8–26%)	♀: 11% (2–18%)	♀: 6% (3–12%)	♀: 12% ( 7–21%)		
**Endometrial**	~3%	30–35%	15% (6–35%)	12% (3–20%)	10% (5–17%)	14% (7–24%)	N/A	
**Ovarian**	~1%	6–14 %	N/A	SIR: 12.0 (3.3–30.7)	HR: 1.52 (0.45–5.05)			
**Gastric**	~1%	0.7–13 %		SIR: 0.0 (0–6.5)	HR: 2.07 (0.73–5.87)			
**Urothelial**	~1–2%	1.9–11.2%		SIR (bladder): 2.0 (0.05–11.2)	HR: 2.05 (0.77–5.45) (kidney and ureter)			
				SIR (renal pelvis): 50.5 (6.1–182.4)				
**Small Bowel**	~0.1%	0.6–7%		SIR: 118.9 (38.6–277.4)	Too few events for analysis			
**CNS**	~0.5%	1.2–3.7%		SIR: 2.7 (0.069–15.2)	HR: 2.09 (0.79–5.54) (brain)			
**Pancreas & biliary tree**	~1–2%	0.6–2.1%		SIR: 0 (0–12) (only pancreas)	HR: 1.02 (0.12–8.60) (hepatobiliary)			
**Breast**	~12%	Conflicting results of association		SIR: 3.8 (1.9–6.8)	HR: 1.30 (0.79–2.16)			

SIR: Standardized Incidence Ratio. HR: Hazard Ratio. 95% CI: Confidence Interval.

IMRC: International Mismatch Consortium. PLSD: Prospective Lynch Syndrome Database.

*path_MLH1, path_MSH2 and path_MSH6 combined.

All three of the studies described above used modified segregation analysis to correct for ascertainment bias. This form of bias is the selection of families with relatively high penetrance due to selection criteria, most likely resulting in overestimation of cancer risk (24). However, retrospective analyses have been important because they estimate risk without surveillance. But there are other ways to deal with ascertainment bias besides modified segregation analysis. The first is to include families that have not been ascertained because of the LS phenotype, a group that includes those ascertained through universal IHC for the MMR proteins. These families usually exhibit much milder phenotypes compared to clinically ascertained families. A recent study published on MedRxiv reported much lower cancer risks and families ascertained through population screening in comparison to clinical ascertainment, namely 15.2% vs. 27.1% for *path_MLH1* and 3.2% vs. 25.2% for *path_MSH2*, respectively (25). Theoretically this could mean that *path_PMS2* carriers ascertained from the population or as incidental findings would only be at population risk (see also the clinical challenges section in this review). Cancer risks established in this way would be of high value considering the occurrence of *path_PMS2* variant detection as incidental findings for example (see case discussions below). However, such cases mostly remained unidentified before the introduction of universal screening and to our knowledge such data is currently unavailable in sufficient quantities to estimate these risks.

Ascertainment bias may also be circumvented by analysing families ascertained because of a patient with CMMR-D. These patients carry homozygous or compound heterozygous variants in one of the MMR genes, usually *PMS2* or *MSH6*. Due to their constitutional dMMR, these patients display a very striking phenotype of cancer in childhood. They also present with axillary freckling and café-au-lait macules (19, 26–29). As *de novo* MMR variants have been reported to be extremely rare, parents are usually carriers of a heterozygous MMR variant, and therefore have LS. Notably, these families almost never meet traditional selection criteria due to a very mild phenotype. Our group has gathered a large cohort of CMMR-D-ascertained LS families and found similar results to the previously published cancer risks, 8.7% (95% CI 4.3–12.7%) up to age 70 years for both sexes combined (30), which confirms previous reports of low cancer risks for *PMS2*-LS patients. The great advantage of this method is that it provides cancer risks similar to the retrospective approach, i.e. risks without surveillance, but without ascertainment bias of families selected based on the LS phenotype. However, due to the rarity of families with a CMMR-D case the amount of families that can be included is much lower.

A third method of estimating cancer risks is to gather prospective data, and a global collaboration has now been formed to gather such data. The Prospective LS Database (PLSD) has currently published multiple reports on penetrance (23, 31–33). Their most recent study estimated risks of 11% (95% CI: 3–37%) and 8% (95% CI: 2–29%) for *path_PMS2* carriers up to age 70 for men and women, respectively (32). Cancer risk estimates from the PLSD are aimed at determining the risks while under surveillance, which is important information when counselling patients. However, comparison of these risks to retrospective studies are difficult due to the inherent differences of these approaches. For a further discussion on this we refer to the section on clinical guidelines.

Lastly, it is well known that cancer penetrance seems to vary between and within families. One study that attempted to capture this variation by estimating the proportion of carriers that were at a specific risk found that most *path_PMS2* carriers had a cumulative risk lower than 20%. However, a small fraction of carriers were still at very high risk (more than 80%) (34). It is likely that other (strong) risk factors play a role in these carriers and/or families, as discussed in more detail below.

### Genotype–phenotype correlations

One factor that could influence penetrance is the specific variant present. Whether or not cancer penetrance in LS patients is dependent on the specific type of pathogenic variant identified in an MMR gene is still a subject of debate. It is conceivable that variants that lead to a partially functional protein could explain more mildly affected families. However, a recent PLSD report found no differences in penetrance between missense or truncating variants in *path_MLH1* or *path_MSH2* carriers (35). Missense and truncating mutations make up the majority of reported *path_MLH1* (both 40%), *path_MSH2* (31% and 49%, respectively), and *path_MSH6* (49% and 43%, respectively) variants. In contrast, missense mutations make up 62% of *path_PMS2* variants, considerably higher than the percentage of truncating mutations (24%) (36). There is one study of European *path_PMS2* carriers that identified a difference of 9 years delay in mean age at first CRC diagnoses for variants that had retained RNA expression (37). Based on this study, most of the variants discovered were categorized as missense variants causing the loss of *PMS2* mRNA expression. There were also several missense variants that did not seem to impact *PMS2* mRNA expression (i.e. c.137G>T (p.Ser46Ile) and c.2113G>A (p.Glu705Lys)), which resulted in residual function of PMS2 protein. The residual protein function might explain the fact that this group of patients develop CRC at older age compared to the group bearing variants affecting mRNA expression (51.1 years vs. 60 years, respectively). Of note, effects on RNA expression could not be taken into account in the PLSD study because these data were not available. So whether these findings can also be extrapolated to other MMR genes remains to be determined. Interestingly, a report on CMMR-D patients carrying a biallelic NM_000535.5:c.2002A>G (p.Ile668Val) variant described an attenuated phenotype where the age at first cancer was strikingly different, namely 22 years for carriers of this variant versus 8 years for truncating *PMS2* variants (38). Functional studies in these patients showed they retained full-length protein in normal tissue. These findings, if replicated, could have important consequences for clinical risk stratification and even surveillance guidelines.

### Molecular pathways of dMMR-associated carcinogenesis

In healthy individuals, MMR proteins function as heterodimers in two main complexes consisting of (1) MutS homologues MSH2 and either MSH6 or MSH3 and (2) MutL homologues MLH1 binding to PMS2, PMS1 or MLH3. The MutS complex recognises a mismatch between the opposing DNA strands and recruits the MutL complex, which then initiates repair. These complexes act together in repairing mismatches of single nucleotides and insertion-deletion loops (5, 36, 39). The theory regarding the lower penetrance of *path_PMS2* variants is that MLH1/MLH3 and/or MLH1/PMS1 heterodimers can partially compensate for the loss of the MLH1/PMS2 heterodimer. Indeed, *Pms2* -/- and *Mlh3* -/- mice have a similar mutational load and disease progression to *Mlh1* -/- mice, suggesting this is a plausible explanation (40).

Tumours in *path_MMR* carriers arise or progress when the remaining wild type *MLH1*, *MSH2*, *MSH6*, or *PMS2* allele is deactivated because of a second hit, in line with Knudson’s “Two-hit” hypothesis (41). This leads to impaired MMR and subsequent accumulation of somatic variants in other (cancer) genes, which can eventually lead to uncontrolled cell growth and cancer. Hallmarks of these tumours are the absence of MMR protein expression by IHC and, as a result of faulty MMR, the shortening and lengthening of regions with nucleotide repeats. These regions, which are common in our genome, can exist inside and outside protein coding regions and are referred to as microsatellites, with changes in their length referred to as microsatellite instability (MSI). Although these changes can also be caused by somatic pathogenic MMR gene variants when they hit both alleles of a MMR gene, they are very helpful in the selection of patients for germline DNA testing for LS (42).

**Figure 1:**
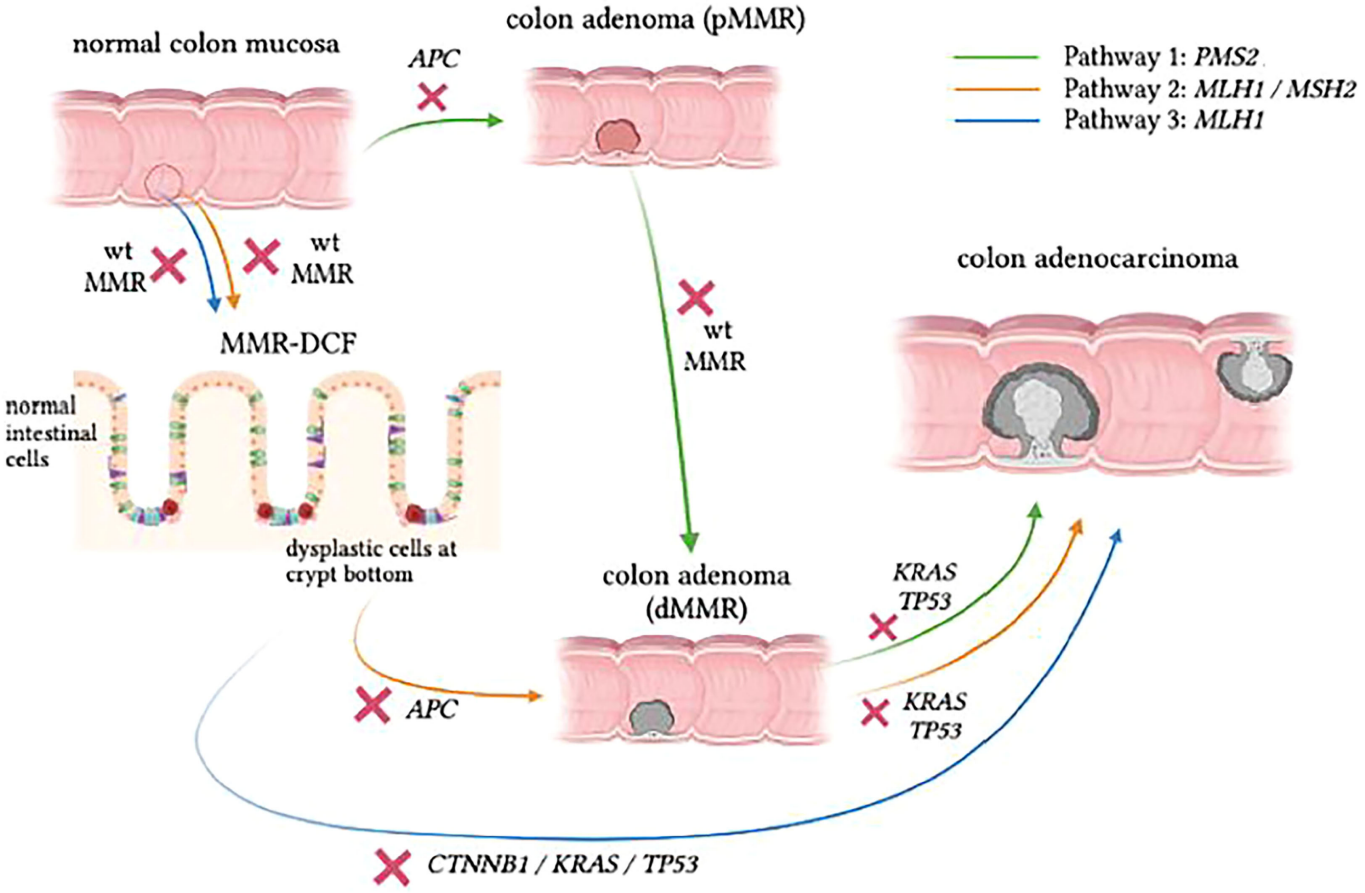
Proposed pathways of CRC development in LS. Pathway 1 (green) shows similarities to the classic adenoma-carcinoma sequence, but the progression speed is accelerated in LS carriers. Pathway 2 (orange) follows the pattern of adenoma formation from MMR-DCF, which eventually gives rise to adenocarcinoma as a consequence of accumulating somatic mutations with absent MMR activity in the background . In Pathway 3 (blue), which is rather insidious, dMMR LS-CRC can skip the adenomatous phase to grow directly into the colonic wall. Figure based on data from Ahadova et al., 2018[42] and Engel et al., 2020[68].

Testing for MSI, somatic variants caused by MSI, or other mechanisms and loss of MMR by IHC staining also plays a role in the study of LS carcinogenesis. Recent studies have proposed three distinct carcinogenesis pathways in LS (**Figure 1**) (43, 44). These are:

Pathway 1 – the traditional proficient MMR (pMMR) adenoma to dMMR CRC pathway,
Pathway 2 – a combined pathway where dMMR adenomas grow from dMMR crypts proceeding to dMMR CRC and
Pathway 3 – a more recently discovered pathway where dMMR CRC develops directly from morphologically normal dMMR crypt foci (MMR-DCF).

About a decade ago, IHC staining of colon specimens from LS patients revealed areas of morphologically normal mucosa that were already devoid of MMR protein, which demonstrated early loss of MMR function in otherwise normal-looking mucosa (45). These areas are referred to as mismatch repair deficient crypt foci (MMR-DCF), and they are considered unique to LS because they are rarely found (<1%) in biopsy specimens of normal mucosa in the vicinity of sporadic MSI-high tumours (45). In addition, MMR-DCF were more frequently found in colon tissue than in small intestine in LS patients, and their abundance might increase with age (46). Most likely, a large percentage of LS-CRC cases develop through MMR-DCF that continuously accumulate variants, resulting in malignant transformation of colonic cells regardless of the presence of an adenoma, as indicated in **Figure 1**. In the past, somatic β-catenin variants have been linked to CRC formation from MMR-DCF (47). The finding that β-catenin variants have not been observed in PMS2-LS-CRC has led to the hypothesis that CRCs in these *path_PMS2* carriers developed through an adenoma precursor lesion and not directly from MMR-DCFs (48).

A possible explanation for the differences observed between *path_PMS2* carriers and other *path_MMR* carriers is that dPMS2 may only occur at a later stage of tumour development (Pathway 1). More information on the specific mutational spectrum of LS-tumours could help to further corroborate this. Somatic variants identified in LS-lesions often demonstrate an overrepresentation of C>T variants, which corresponds to mutational signature 6 associated with MMR deficiency (48, 49). Mutated genes in dMMR CRC include *APC*, *KRAS*, *CTNNB1* and *TGFBR2*. Somatic *APC* variants are assumed to occur after the loss of the wildtype MMR allele in the majority of LS-CRCs and to accelerate the malignant transformation in LS. However, although the majority of dysplastic LS adenomas are dMMR, it is important to note that some adenomas in LS do retain MMR capacity (pMMR adenomas), in accordance with Pathway 1 (43). It has also been suggested that dPMS2 CRC develops solely through pMMR adenomas, with dPMS2 occurring at a relatively late stage and not as an initiating event. Indeed, data from a previous study by our group reported a relatively low frequency of the somatic *KRAS* hotspot variants G12D and G13D that were previously associated with the mutational signature of MMR deficiency (48, 49). The lower frequency of these two variants in dPMS2 tumours, combined with the fact that *KRAS* variants are known to occur in a relatively late stage of tumour progression, has led to the hypothesis that loss of the wildtype *PMS2* allele is a secondary and not an initiating event in CRCs that develop in *path_PMS2* carriers. Future studies are needed to confirm this hypothesis and evaluate why dPMS2 predominantly contributes as a late event in LS carcinogenesis.

Another approach to investigate differences between molecular pathways in the different subgroups of *path_MMR* carriers is to study the coding microsatellite instability (cMSI) spectrum (50). Our group therefore performed a second tumour study analysing 16 dPMS2 CRCs from confirmed *path_PMS2* carriers. The cMSI spectrum of dPMS2 CRCs did not show any significant differences from dMLH1/dMSH2 CRCs, even after correction for tumour stage. If confirmed by larger studies, this is an interesting finding from an immunological perspective. An important aspect of dMMR CRC is activation of the immune system. CRCs in *path_MMR* carriers are known to bear a significantly higher number of pathogenic variants compared to sporadic CRC. These lesions with high mutational burden produce a relatively large amount of tumour-specific neoantigen. In the case of LS-CRC, the neoantigens resulting from insertions and deletions occurring in tumour cells with MSI are caused by a shift of the translational reading frame, with the resulting neoantigens termed frameshift peptides (FSPs). The presence of FSPs on the cellular membrane can trigger the recruitment and functionality of immune cells that surround the tumour mass. Consequently, MSI tumours display a high degree of immune infiltration. This is considered to play a major role in the favourable prognosis of LS-tumours (23). However, we and others have observed significantly lower CD3-positive T cell infiltration in dPMS2-CRCs compared to other dMMR-CRCs. One study looked at 93 dPMS2-CRCs and observed higher odds of disease specific death compared to other dMMR-CRCs. A plausible explanation for this more aggressive behaviour may be the lower degree of immune activation. The same study speculated that a lower level of mutational neoantigens may underlie the limited T cell infiltration in dPMS2 tumours (51). As described above, our data did not provide any evidence for a decreased amount of cMSI–induced neoantigens in dPMS2 CRCs. Therefore other explanations should be considered, including alternative immune evasion strategies, similar to what is seen in sporadic CRC. Moreover, these findings may be compatible with the hypothesis that dPMS2 occurs later during tumour evolution.

How do these MMR gene-dependent pathways relate to EC? Recent work has shown the existence of dMMR nonneoplastic endometrial glands (52). These dMMR glands were not present in population controls, suggesting that they are a benign precursor for EC in LS patients. More studies are needed to determine whether there are multiple pathways leading to EC and whether or not there are differences between the MMR genes as well.

### Clinical guidelines

Interestingly, the CRC risk reported for *path_PMS2* carriers is only two or maximum three times higher than the general population risk. Is that high enough to offer surveillance colonoscopy? In the Netherlands for example, surveillance would be indicated when the CRC risk exceeds the threshold of three times the general population risk. Does it therefore follow that *path_PMS2* carriers should not undergo any colonoscopic surveillance, at least in countries using these threshold levels? To answer this question, we can look at several lines of evidence.

Firstly, a recent study compared retrospective International Mismatch Repair Consortium (IMRC) and prospective cancer risks from the PLSD (32). Retrospective studies are aimed at determining the cancer risk without colonoscopy and polypectomy, while prospective studies include patients that undergo surveillance and therefore estimate risk despite colonoscopy. The retrospective CRC risks for *path_PMS2* carriers were very similar, 7% for men (95% CI: 6–8%) vs 6% for women (95% CI: 5–6%), while the prospective risk was 11% (95% CI: 3–37%) vs 8% (95% CI: 2–29%). However, before age 50, the prospective *path*_*PMS2* PLSD cohort appeared to have slightly lower CRC risk than the retrospective IMRC cohort, although this was not statistically significant. Of note, this was the opposite for carriers of other *path_MMR* variants, suggesting that colonoscopic surveillance does not prevent CRC in these carriers, but might in fact be effective for *path_PMS2* carriers. A possible explanation for this is that dPMS2 tumours are believed to predominantly progress from adenomas, which are clearly visible, while the other dMMR tumours may progress directly from MMR-DCF, which may be more difficult to detect during colonoscopies. In the future, larger studies should shed more light on whether this is a clinically relevant difference. This naturally has important implications for the determination of clinical guidelines.

Secondly, as discussed above, the role of dPMS2 in tumour development seems to be confirmed by molecular studies and could result in increased adenoma progression to CRC (50). For the moment, the clinical and molecular characteristics together support the existence of colonoscopy guidelines for *path_PMS2* carriers. Whether use of other screening measures such as the Fecal Immunochemical Test instead of colonoscopy might also lead to substantially (and acceptably) lower cancer risk needs further research.

## Present

### Challenges in clinical practice

Recent guidelines have made a clear distinction between the different MMR carriers (53–56). For *path_PMS2* carriers colonoscopic surveillance starts at age 35 rather than at age 25. The foundation for this being the substantially lower cancer risk and later age at onset of CRC compared to other *path_MMR* carriers, as discussed above. Indeed, recent studies have shown that raising the starting age is (very) cost-effective without leading to substantial differences in disease outcomes (57, 58). The European Hereditary Tumour Group guideline takes this one step further by extending the colonoscopy interval to 5 years from 1–2 years (56). Below we present and discuss five cases from our daily practice that highlight challenges in clinical management of *path_PMS2* carriers. The aim of presenting these cases is not to replace current clinical guidelines but to serve as an illustration and stimulate further discussion.

### Case 1

The daughter of a 60-year-old woman who had died from endometrioid type ovarian cancer is referred for genetic testing. Unfortunately, tumour tissue from the mother is not available for sequencing and/or IHC. In line with current Dutch guidelines, the daughter is offered germline DNA testing of our ovarian cancer gene panel, which includes the MMR genes. A likely pathogenic germline variant in the *PMS2* gene is identified. There is no personal or family history of EC or CRC.

*Case discussion:* We suggest counselling the daughter and explaining that there is no evidence for a direct association between pathogenic variants in this gene and ovarian cancer (see also **Table 1**). The penetrance of this variant is likely very low given that the family history mentions no LS tumours. We believe that the *path_PMS2* variant should therefore be considered an incidental finding and could have been inherited from the father or mother. The pros and cons of colonoscopic and endometrial surveillance should carefully be discussed with the daughter in light of the likely low penetrance. Unfortunately, cancer risk data in such instances are not yet available.

In our centre we have now excluded the *PMS2* gene from the ovarian cancer panel because of lack of evidence for an association. Germline testing of this gene in an ovarian cancer panel would, in our opinion, be opportunistic screening, i.e. aimed at finding genetic disease predisposition unrelated to the diagnostic question. Such screening is currently not offered to our patients who undergo diagnostic testing.

### Case 2

A ten-year-old boy from two non-consanguineous healthy parents presents with severe developmental delay and dysmorphic features. A SNP array is performed, and a small paternal deletion identified that includes 7p22.1 where the *PMS2* gene is located. It is believed that this is not an explanation for the boy’s developmental delay and whole exome sequencing will be performed next. There is no family history of cancer of any type and no consanguinity. Should the pathogenic deletion of *PMS2* be discussed as an incidental finding and cascade screening subsequently be offered?

*Case discussion:* Further policy in this case is naturally highly dependent on national guidelines and the specific informed consent given by the parents regarding incidental/secondary findings. In the Netherlands, the standard is to only report highly penetrant variants in genes with clinical actionability. Based on UK Biobank findings for *MLH1* and *MSH2 (* 25), we suspect that *path_PMS2* cancer risks in the general population are even lower than reported for *PMS2*-LS families and might fall well below the national threshold of three times the population CRC risk, therefore not warranting such surveillance. Nevertheless, these risk figures are unavailable, and the only national *PMS2* guideline available to us is that for identifying pathogenic variants in the setting of suspected LS, which recommends colonoscopic surveillance. The ACMG guidelines on reporting secondary findings in patients undergoing diagnostic testing *do* recommend actively looking at *PMS2* for pathogenic variants and reporting those even if unrelated to the disease for which the testing was initially done (59). Those guidelines, however, are often based on cancer risk studies biased by selection and ascertainment, and this holds true for *PMS2*. A last argument in favour of reporting the deletion in this setting could be the identification of couples at risk of conceiving a child with CMMR-D. However, to our knowledge in most countries genetic testing for *path_MMR* variants is not routinely offered to partners of *path_MMR* carriers who want to conceive. The exception being cases with known consanguinity or a higher chance of biallelic offspring (e.g. isolated populations). Clearly there are pros and cons for reporting our *PMS2* finding in such situations: the possible increased CRC risk on the one hand and the lack of knowledge of cancer risks associated with *path_PMS2* as secondary finding, which might in fact be low, on the other. In the end we decided to share these considerations with the parents in addition to discussing the burden of colonoscopy and the possible alternative of the national CRC screening through faecal occult blood testing.

### Case 3

A 30-year-old woman is pre-symptomatically tested for the *path_PMS2* variant in her family and found to be positive. She requests a prophylactic hysterectomy and oophorectomy. There is no family history of ovarian cancer and EC.

*Case discussion:* We advise extreme restraint with respect to a prophylactic hysterectomy in this case. The cumulative risk of EC is approximately 12% (3). Moreover, as survival for these tumours is extremely high (23, 33), we believe there is no indication for prophylactic hysterectomy. Of note, the Manchester recommendations for the management of gynaecological cancer in LS involved patient representatives who felt that it should be considered an option despite these considerations (53). However, in the Netherlands, we currently actively advise against gynaecological preventive surgery in *path_PMS2* carriers.

### Case 4

A 55-year-old man presents with a T3N0 CRC, with no family history of CRC or EC. Universal IHC for the MMR proteins shows loss of expression of the PMS2 protein. The surgeon refers the patient for priority counselling and genetic testing. Choice of a specific operating procedure (i.e. segmental or hemi-colectomy) is postponed until the genetic testing results are available.

*Case discussion:* Extended colectomy with ileosigmoidal/ileorectal anastomosis is preferable to standard resection for *path_MLH1* and *path_MSH2* carriers given the increased risk of developing a metachronous cancer after segmental colectomy vs more extensive surgery (60–62). There is no clear indication for preventive colorectal surgery in *path_PMS2* carriers given the relatively low penetrance of *path_PMS2* variants (56). This means that there is no reason for priority counselling and testing in this case. Decisions regarding the specific operating procedure in this case should be made strictly on patient and tumour characteristics. This has also been included in recent international guidelines (56). The presence or absence of a germline *path_PMS2* variant is not a factor herein.

### Case 5

A 65-year-old woman presents with an MSI CRC and isolated loss of PMS2 expression on IHC. No germline *path_MMR* variant is found, nor is there *MLH1* promoter hypermethylation. There is no family history of CRC or EC. The tumour is sent to the pathology department for next generation sequencing of *PMS2*, where one somatic hit in *PMS2* with a variant allele frequency of 23% is found. There are no signs of loss of heterozygosity indicating loss of the second allele. What would the advice for relatives be in this case?

*Case discussion:* Per definition, the cause of the dMMR in this case was not found. In the past this would mean that first-degree relatives should be offered a LS-like surveillance scheme. We suggest using new techniques to look for a missed germline variant, such as ultra-long-read sequencing to look for deep intronic variants (or exon deletions), which were recently described as an explanation for part of the missing heritability in up to almost 20% of LS-like cases (63). If such analyses also fail to reveal a germline variant, we would not advise additional screening of the colon or endometrium for relatives as the explanation of the dMMR would most likely be somatic. Naturally, relatives would be encouraged to participate in population-based screening. Of note, the possibility of a (missed) germline mosaicism cannot be excluded, but we believe the chance of vertical transmission to offspring can be considered negligible in this case.

## Future

### Sequencing strategies

As mentioned in discussion of Case 5, new methods of MMR gene analyses are now available. Ultra-long-read sequencing with reads up to 100 kb could increase the proportion of LS families identified through more efficient detection of both larger deletions and noncoding, deep intronic variants (63–65). The introduction of these strategies is very relevant for *PMS2* because it makes circumvention of pseudogenes much more straightforward (66).

### Prevention

#### Colonoscopic techniques

There has been a debate about whether standard colonoscopy techniques are adequate to detect all colonic lesions in LS. A previous study in France indicated that optimisation of colonoscopy, such as performing chromoendoscopy, and the adjustment of surveillance intervals led to a significant reduction of CRC incidence (67). According to a recent randomised study, the use of white light endoscopy might aid detection of flat lesions, showing a higher detection rate compared to standard colonoscopy (65% vs. 37% respectively, p 0.003) but detecting comparable numbers of total adenomas and right-sided lesions (68). Unfortunately, flat adenomas, which are presumed to harbour more advanced histology, were still frequently missed by current standard colonoscopy. Given the differences in occurrence of benign precursor stages – MMR-DCF for *path_MLH1*, dMMR adenomas for *path_MSH2* and pMMR adenomas for *path_PMS2* carriers (43, 44) – optimisation of colonoscopy procedures would need to be MMR gene–specific.

#### Chemoprevention

Results from the CAPP2 trial concluded that aspirin intake might prevent nearly half of the CRC diagnosed among individuals affected by LS at low cost and relatively low risk (69). However, there seemed to be a delay in the effect, which only started to be measurable 3–4 years after commencement of chemoprevention, and the effect seemingly occurred when aspirin was taken for at least 2 years. At present the precise mechanism of aspirin in cancer reduction is not known. One hypothesis is modulation of the immune response that potentially enhances T cell activity while suppressing inflammatory responses. An alternative explanation from cell line and mouse model data suggests that aspirin has a pro-apoptotic influence on premalignant cells in the gut, with one conceivable target being MMR-DCF (70). If true, aspirin might have a lower efficacy in *path_PMS2* carriers as they most likely do not develop CRC from MMR-DCF. However, without MMR gene–stratified analyses, this remains speculation.

#### Vaccination strategies

In the past few years, increased knowledge about immunotherapeutic approaches has also offered additional cancer-preventive strategies for individuals affected by LS. Recent studies revealed that FSP-specific immune responses were already detectable in tumour-free *path_MMR* carriers or individuals with early-stage adenomas, indicating that continuous immunoediting occurs in early LS lesions (71). FSPs derived from cMS variants in dMMR cells can be processed and presented to the host’s immune system, potentially triggering immune responses specifically targeting dMMR cells. There is evidence that a high mutational and neoantigen load in tumour cells is associated with the strength of antitumoral immune responses. FSP-based preventive vaccination is currently under investigation, and the first phase I/IIA trial has already demonstrated its safety and immunological effectiveness. The therapy regimen consisted of three FSP neoantigens administered subcutaneously using the adjuvant Montanide ISA 51 in three treatment cycles consisting of four weekly applications. Patients demonstrated considerable response to the FSP neoantigens, and the safety profile was deemed tolerable (72). Investigations of cancer-preventive effects of the FSP vaccine in LS carriers remain to be planned, but recent results from a LS mouse model demonstrated that FSP vaccines can reduce tumour burden and improve survival (73). The presence of a similar cMSI spectrum, and thus of FSPs for dPMS2 tumours (as compared to dMLH1 or dMSH2 tumours), could be reassuring for the expected efficacy of FSP-based vaccines (50). However, the effect of lower immune infiltration remains to be seen. The presence of pMMR adenomas rather than MMR-DCF as a benign precursor in *path_PMS2* carriers could mean that FSPs are presented at a later stage, potentially resulting in a lower efficacy for this type of vaccine. This underlines the need for MMR gene–stratified studies of tumorigenesis in LS.

#### Immunotherapy

Upregulation of PD-L1 is presumed to be the main immune-evasion mechanism observed in LS-CRC (74, 75). PD-L1 expression has been found in excess in the immune cells at the invasive margin of the tumour bulk as well as the peri- and intra-tumoral macrophages, while its expression on tumour cells was relatively low (76). Multiple studies have now shown the effectiveness of immunotherapy that targets immune checkpoints in dMMR CRC (77–79). PMS2-deficient CRC is unique since it is associated with fewer immune features, such as less pronounced CD3+ T cell infiltration in the tumour milieu (48, 51). This finding indicates that these tumours might develop a lesser degree of immune activation, leading to more aggressive tumour behaviour. It is plausible that dPMS2 tumours use strategies typical of pMMR CRCs to avoid the immune system. However, whether these findings impact the effectiveness of immunotherapy of dPMS2-CRC is still debatable because immune profiling studies that focus on dPMS2-CRC remain scarce. This is an area that needs to be explored further as it could have significant impact on the clinical management of these patients.

### Risk-modifying factors

#### Microbiome

The human intestine is colonised by various types of resident microorganisms, including bacteria, archaea, viruses and fungi, which make up the diverse human microbiota. The impact of dysbiosis in carcinogenesis in the LS population has just begun to be explored. One study has demonstrated a different stool microbiota composition between healthy carriers of LS and LS-CRC patients (80). Another study concluded that stool samples of LS patients with adenoma showed lower amounts of *Clostridiaceae* and increased amounts of *Lachnospiraceae, Desulfovibrio* sp. and *Ruminococcaceae*. Interestingly, this study also revealed that the underlying germline *path_MMR* variant also affected the microbial species observed. A decreased amount of *Blautia* sp. was observed in *path*_*MLH1* and *path*_*MSH2* carriers, while an enriched abundance of *C. bartletti* and *Alistipes* sp. was observed in samples from *path_PMS2* carriers (81). From the results described above, it is evident that our understanding of the reciprocal association between gut dysbiosis and LS-CRC development is far from complete. Complex *in vitro* culturing models such as organoids and organ-on-a-chip will aid in answering these questions, including a delineation of cause versus consequence. It is also conceivable that different microorganisms play a role at different stages of tumour evolution, which is why it is also extremely relevant to take the different pathways of CRC development into account. For example, the microbiome could have an important influence on the development of pMMR adenomas, which are most likely the benign precursors for CRCs in *path_PMS2* carriers, as described above. The gut microbiome therefore has potential to be a controllable factor in *path_PMS2* carriers.

#### Lifestyle factors

Lifestyle factors such as smoking habits, physical activity and BMI have been associated with modification of CRC risk among *path_MMR* carriers (82, 83). Previous studies have firmly established the correlation between dietary intake of certain nutrients and increased risk of intestinal inflammation (84, 85). The GeoLynch study conducted in the Netherlands further confirmed that diets rich in processed meat, sugar and refined grains were thought to support the inflammatory process in the gut, which might eventually promote carcinogenesis in LS-CRC (86). In contrast, diets rich in fibre, vegetables, legumes and fish were associated with a reduced risk of sporadic CRC formation. Recently, a study on the preventive use of resistant starch in LS patients showed no impact on CRC risk but did find a significant reduction in extra-colonic cancer (87, 88). Since most patients were *path_MLH1* (60%) or *path_MSH2* carriers (37%), with only a few *path_MSH6* (3%) and no *path_PMS2* carriers, the effect of the use of resistant starch in these two last groups remains a question. Previously studied cohorts were also underpowered to look at MMR gene–specific effects of lifestyle factors. Whether improving the lifestyle of LS carriers would confer a later age of CRC onset, milder type of disease, or no CRC manifestation at all also still needs to be addressed.

While results are somewhat conflicting, most studies find no differences in risk profiles including BMI, co-morbidity and lifestyle factors between sporadic and LS-EC (89). For example, higher BMI seems to increase EC risk in both groups. In contrast, some hormonal factors appear to have a risk-lowering effect, although here again there was no difference between sporadic and LS-EC (90). This suggests that these factors might not have MMR gene–specific effects, but future research should shed more light on this.

### Digenic inheritance of pathogenic variants in other genes

The reduced penetrance of a pathogenic variant might be caused by either genetic or non-genetic factors, collectively influencing disease manifestation. Presence of disease-modifying variants or the occurrence of different somatic variants might explain the varying penetrance of the germline variant, since less common germline variants in the human genome may modify the expression of major genes. Digenic inheritance is the phenomenon in which presence of genetic variants (inherited or arising *de novo*) impacts both the penetrance of the gene of interest and the observed phenotype. It further emphasises the complex genotype–phenotype interactions (91). Digenic inheritance is considered a major contributor to the variable phenotypes observed in hereditary disorders, which might modify predisposition to illness or cause heterogeneity in disease manifestation among family members. Digenic inheritance has also been described in Lynch syndrome (92). The facts that *path_PMS2* carriers do not develop cancer as frequently as other *path_MMR* carriers and members of the same family affected by the same *path_PMS2* variant also demonstrate a wide range of age for cancer-onset offer potential evidence of digenic inheritance among this group of carriers. Indeed, a very recent case-report described two teenage siblings with multiple adenomas and CRC with a maternally inherited *path_PMS2* variant and a paternally inherited *path_POLD1* variant (93). Interestingly, molecular studies of the tumours revealed an ultra-mutated tumour phenotype with mutational signatures of both *PMS2* and *POLD1*, suggesting that these factors interacted to cause the relatively severe clinical phenotype in these cases.

Efforts to discover other potential genetic modifiers or epigenetic events that could contribute to LS manifestations, especially among *path_PMS2* carriers, have to be pursued as they have evident consequences for clinical management.

### Influence of HLA genotype

As described above, development of CRC is influenced by activation of the immune system. The presence of immunogenic FSPs has the potential to activate the immune system and thereby prevent early dMMR lesions from progressing to CRC. A key factor in the presentation of FSPs to immune surveillance is the HLA complex. This has led to the hypothesis that HLA genotype could be an explanation for the observed differences in phenotype, i.e. differences in cancer risks and age of onset. No data on this is currently available, but a new initiative has been established to further investigate this (INDICATE, http://indicate-lynch.org/) (94). Analysing such data in a MMR gene–stratified manner seems critical given the differences in preferential carcinogenic pathways. Hypothetically, the presence of pMMR adenomas as the benign precursor of CRC in *path_PMS2* carriers could mean that the HLA genotype is not as strong a predictor for cancer risk in these carriers because of the absence of FSPs in early lesions, which could be targeted by early immune surveillance.

## Conclusion

In the past decade it has become apparent that clinical and biological characteristics of LS patients are highly dependent on the specific MMR gene affected. PMS2-LS clearly represents the milder end of the phenotypic spectrum and has its own unique pathophysiology. Surveillance guidelines now recommend a starting age of 35 years for colonoscopy for *path_PMS2* carriers and take a (very) conservative approach towards gynaecological surveillance and, more specifically, prophylactic surgery. While the cancer risks are relatively low, as dPMS2 most likely occurs later in tumour development, these tumours share characteristics with dMMR CRC as well as sporadic pMMR CRC. This could result in clinically important differences with regard to (chemo)prevention and therapy. The improvement of fundamental research techniques and increased detection of *path_PMS2* carriers will lead to a more thorough understanding of this specific subset of LS patients and aid in clinical decision-making in the future.

## Author contributions

KA, MN, KK, RS and SB contributed to conception and design of the review. KA and SB wrote the first draft of the review. All authors contributed to the article and approved the submitted version.

## References

[1] Win AK Jenkins MA Dowty JG Antoniou AC Lee A Giles GG. Prevalence and penetrance of major genes and polygenes for colorectal cancer. Cancer Epidemiol Biomarkers Prev (2017) 26(3):404–12. doi: 10.1158/1055-9965.EPI-16-0693 PMC5336409 27799157

[2] Senter L Clendenning M Sotamaa K Hampel H Green J Potter JD. The clinical phenotype of lynch syndrome due to germ-line PMS2 mutations. Gastroenterology (2008) 135(2):419–28. doi: 10.1053/j.gastro.2008.04.026 PMC2759321 18602922

[3] Ten Broeke SW van der Klift HM Tops CMJ Aretz S Bernstein I Buchanan DD. Cancer risks for PMS2-associated lynch syndrome. J Clin Oncol (2018) 36(29):2961–8. doi: 10.1200/JCO.2018.78.4777 PMC6349460 30161022

[4] ten Broeke SW Brohet RM Tops CM van der Klift HM Velthuizen ME Bernstein I. Lynch syndrome caused by germline PMS2 mutations: delineating the cancer risk. J Clin Oncol (2015) 33(4):319–25. doi: 10.1200/JCO.2014.57.8088 25512458

[5] Peltomaki P. Role of DNA mismatch repair defects in the pathogenesis of human cancer. J Clin Oncol (2003) 21(6):1174–9. doi: 10.1200/JCO.2003.04.060 12637487

[6] Nicolaides NC Papadopoulos N Liu B Wei YF Carter KC Ruben SM. Mutations of two PMS homologues in hereditary nonpolyposis colon cancer. Nature (1994) 371(6492):75–80. doi: 10.1038/371075a0 8072530

[7] van der Klift HM Tops CM Bik EC Boogaard MW Borgstein AM Hansson KB. Quantification of sequence exchange events between PMS2 and PMS2CL provides a basis for improved mutation scanning of lynch syndrome patients. Hum Mutat (2010) 31(5):578–87. doi: 10.1002/humu.21229 20186688

[8] van der Klift HM Mensenkamp AR Drost M Bik EC Vos YJ Gille HJ. Comprehensive mutation analysis of PMS2 in a large cohort of probands suspected of lynch syndrome or constitutional mismatch repair deficiency syndrome. Hum Mutat (2016) 37(11):1162–79. doi: 10.1002/humu.23052 27435373

[9] Clendenning M Hampel H LaJeunesse J Lindblom A Lockman J Nilbert M. Long-range PCR facilitates the identification of PMS2-specific mutations. Hum Mutat (2006) 27(5):490–5. doi: 10.1002/humu.20318.Erratumin:HumMutat 16619239

[10] Vasen HF Möslein G Alonso A Aretz S Bernstein I Bertario L. Recommendations to improve identification of hereditary and familial colorectal cancer in europe. Fam Cancer. (2010) 9(2):109–15. doi: 10.1007/s10689-009-9291-3 19763885

[11] Clendenning M Senter L Hampel H Robinson KL Sun S Buchanan D. A frame-shift mutation of PMS2 is a widespread cause of lynch syndrome. J Med Genet (2008) 45(6):340–5. doi: 10.1136/jmg.2007.056150 PMC4339871 18178629

[12] Truninger K Menigatti M Luz J Russell A Haider R Gebbers JO. Immunohistochemical analysis reveals high frequency of PMS2 defects in colorectal cancer. Gastroenterology (2005) 128(5):1160–71. doi: 10.1053/j.gastro.2005.01.056 15887099

[13] Hampel H Frankel WL Martin E Arnold M Khanduja K Kuebler P. Feasibility of screening for lynch syndrome among patients with colorectal cancer. J Clin Oncol (2008) 26(35):5783–8. doi: 10.1200/JCO.2008.17.5950 PMC2645108 18809606

[14] Baudhuin LM Burgart LJ Leontovich O Thibodeau SN. Use of microsatellite instability and immunohistochemistry testing for the identification of individuals at risk for lynch syndrome. Fam Cancer. (2005) 4(3):255–65. doi: 10.1007/s10689-004-1447-6 16136387

[15] Halvarsson B Lindblom A Rambech E Lagerstedt K Nilbert M. The added value of PMS2 immunostaining in the diagnosis of hereditary nonpolyposis colorectal cancer. Fam Cancer. (2006) 5(4):353–8. doi: 10.1007/s10689-006-0005-9 16817031

[16] Eikenboom EL van der Werf-'t Lam AS Rodríguez-Girondo M Van Asperen CJ Dinjens WNM Hofstra RMW. Universal immunohistochemistry for lynch syndrome: A systematic review and meta-analysis of 58,580 colorectal carcinomas. Clin Gastroenterol Hepatol (2022) 20(3):e496–507. doi: 10.1016/j.cgh.2021.04.021 33887476

[17] Pearlman R Haraldsdottir S de la Chapelle A Jonasson JG Liyanarachchi S Frankel WL. Clinical characteristics of patients with colorectal cancer with double somatic mismatch repair mutations compared with lynch syndrome. J Med Genet (2019) 56(7):462–70. doi: 10.1136/jmedgenet-2018-105698 PMC6748629 30877237

[18] Haraldsdottir S Rafnar T Frankel WL Einarsdottir S Sigurdsson A Hampel H. Comprehensive population-wide analysis of lynch syndrome in iceland reveals founder mutations in MSH6 and PMS2. Nat Commun (2017) 8:14755. doi: 10.1038/ncomms14755 28466842 PMC5418568

[19] Herkert JC Niessen RC Olderode-Berends MJ Veenstra-Knol HE Vos YJ van der Klift HM. Paediatric intestinal cancer and polyposis due to bi-allelic PMS2 mutations: case series, review and follow-up guidelines. Eur J Cancer. (2011) 47(7):965–82. doi: 10.1016/j.ejca.2011.01.013 21376568

[20] Adar T Rodgers LH Shannon KM Yoshida M Ma T Mattia A. Universal screening of both endometrial and colon cancers increases the detection of lynch syndrome. Cancer (2018) 124(15):3145–53. doi: 10.1002/cncr.31534 29750335

[21] Ryan E Sheahan K Creavin B Mohan HM Winter DC. The current value of determining the mismatch repair status of colorectal cancer: A rationale for routine testing. Crit Rev Oncol Hematol (2017) 116:38–57. doi: 10.1016/j.critrevonc.2017.05.006 28693799

[22] van Lier MG Leenen CH Wagner A Ramsoekh D Dubbink HJ van den Ouweland AM. Yield of routine molecular analyses in colorectal cancer patients ≤70 years to detect underlying lynch syndrome. J Pathol (2012) 226(5):764–74. doi: 10.1002/path.3963 22081473

[23] Møller P Seppälä T Bernstein I Holinski-Feder E Sala P Evans DG. Cancer incidence and survival in lynch syndrome patients receiving colonoscopic and gynaecological surveillance: first report from the prospective lynch syndrome database. Gut (2017) 66(3):464–72. doi: 10.1136/gutjnl-2015-309675 PMC5534760 26657901

[24] Vos JR Hsu L Brohet RM Mourits MJ de Vries J Malone KE. Bias correction methods explain much of the variation seen in breast cancer risks of BRCA1/2 mutation carriers. J Clin Oncol (2015) 33(23):2553–62. doi: 10.1200/JCO.2014.59.0463 PMC4979233 26150446

[25] Jackson L W. M Harrison JW Wood AR Ruth KS Tyrrell J. Influence of family history on penetrance of hereditary cancers in a population setting. MedRxiv (2022). doi: 10.1101/2022.07.08.22277415 PMC10626157 37936660

[26] Bougeard G Olivier-Faivre L Baert-Desurmont S Tinat J Martin C Bouvignies E. Diversity of the clinical presentation of the MMR gene biallelic mutations. Fam Cancer. (2014) 13(1):131–5. doi: 10.1007/s10689-013-9676-1 24068316

[27] Shah KN. The diagnostic and clinical significance of cafe-au-lait macules. Pediatr Clin North Am (2010) 57(5):1131–53. doi: 10.1016/j.pcl.2010.07.002 20888463

[28] Suerink M Potjer TP Versluijs AB Ten Broeke SW Tops CM Wimmer K. Constitutional mismatch repair deficiency in a healthy child: On the spot diagnosis? Clin Genet (2018) 93(1):134–7. doi: 10.1111/cge.13053 28503822

[29] Wimmer K Kratz CP Vasen HF Caron O Colas C Entz-Werle N. Diagnostic criteria for constitutional mismatch repair deficiency syndrome: suggestions of the european consortium 'care for CMMRD' (C4CMMRD). J Med Genet (2014) 51(6):355–65. doi: 10.1136/jmedgenet-2014-102284 24737826

[30] Suerink M Rodríguez-Girondo M van der Klift HM Colas C Brugieres L Lavoine N. An alternative approach to establishing unbiased colorectal cancer risk estimation in lynch syndrome. Genet Med (2019) 21(12):2706–12. doi: 10.1038/s41436-019-0577-z 31204389

[31] Dominguez-Valentin M Sampson JR Seppälä TT Ten Broeke SW Plazzer JP Nakken S. Cancer risks by gene, age, and gender in 6350 carriers of pathogenic mismatch repair variants: findings from the prospective lynch syndrome database. Genet Med (2020) 22(1):15–25. doi: 10.1038/s41436-019-0596-9 31337882 PMC7371626

[32] Møller P Seppälä T Dowty JG Haupt S Dominguez-Valentin M Sunde L. Colorectal cancer incidences in lynch syndrome: a comparison of results from the prospective lynch syndrome database and the international mismatch repair consortium. Hered Cancer Clin Pract (2022) 20(1):36. doi: 10.1186/s13053-022-00241-1 36182917 PMC9526951

[33] Møller P Seppälä TT Bernstein I Holinski-Feder E Sala P Gareth Evans D. Cancer risk and survival in *path_MMR* carriers by gene and gender up to 75 years of age: a report from the prospective lynch syndrome database. Gut (2018) 67(7):1306–16. doi: 10.1136/gutjnl-2017-314057 PMC6031262 28754778

[34] International Mismatch Repair, C. Variation in the risk of colorectal cancer in families with lynch syndrome: a retrospective cohort study. Lancet Oncol (2021) 22(7):1014–22. doi: 10.1016/S1470-2045(21)00189-3 PMC8934577 34111421

[35] Dominguez-Valentin M Plazzer JP Sampson JR Engel C Aretz S Jenkins MA. No difference in penetrance between truncating and Missense/Aberrant splicing pathogenic variants in *MLH1* and *MSH2*: A prospective lynch syndrome database study. J Clin Med (2021) 10(13):2856. doi: 10.3390/jcm10132856 34203177 PMC8269121

[36] Peltomaki P. Update on lynch syndrome genomics. Fam Cancer (2016) 15(3):385–93. doi: 10.1007/s10689-016-9882-8 PMC4901089 26873718

[37] Suerink M van der Klift HM Ten Broeke SW Dekkers OM Bernstein I Capellá Munar G. The effect of genotypes and parent of origin on cancer risk and age of cancer development in PMS2 mutation carriers. Genet Med (2016) 18(4):405–9. doi: 10.1038/gim.2015.83 26110232

[38] Li L Hamel N Baker K McGuffin MJ Couillard M Gologan A. A homozygous PMS2 founder mutation with an attenuated constitutional mismatch repair deficiency phenotype. J Med Genet (2015) 52(5):348–52. doi: 10.1136/jmedgenet-2014-102934 25691505

[39] Jiricny J. The multifaceted mismatch-repair system. Nat Rev Mol Cell Biol (2006) 7(5):335–46. doi: 10.1038/nrm1907 16612326

[40] Jahid S Lipkin S. Mouse models of inherited cancer syndromes. Hematol Oncol Clin North Am (2010) 24(6):1205–28. doi: 10.1016/j.hoc.2010.08.011 PMC3011940 21075289

[41] Knudson AG Jr. Mutation and cancer: statistical study of retinoblastoma. Proc. Natl. Acad. Sci. U S A (1971) 68(4):820–3. doi: 10.1073/pnas.68.4.820 PMC389051 5279523

[42] Umar A. Lynch syndrome (HNPCC) and microsatellite instability. Dis Markers (2004) 20(4-5):179–80. doi: 10.1155/2004/486032 PMC3839338 15528783

[43] Ahadova A Gallon R Gebert J Ballhausen A Endris V Kirchner M. Three molecular pathways model colorectal carcinogenesis in lynch syndrome. Int J Cancer. (2018) 143(1):139–50. doi: 10.1002/ijc.31300 29424427

[44] Engel C Ahadova A Seppälä TT Aretz S Bigirwamungu-Bargeman M Bläker H. Associations of pathogenic variants in MLH1, MSH2, and MSH6 with risk of colorectal adenomas and tumors and with somatic mutations in patients with lynch syndrome. Gastroenterology (2020) 158(5):1326–33. doi: 10.1053/j.gastro.2019.12.032 31926173

[45] Kloor M Huth C Voigt AY Benner A Schirmacher P von Knebel Doeberitz M. Prevalence of mismatch repair-deficient crypt foci in lynch syndrome: a pathological study. Lancet Oncol (2012) 13(6):598–606. doi: 10.1016/S1470-2045(12)70109-2 22552011

[46] Staffa L Echterdiek F Nelius N Benner A Werft W Lahrmann B. Mismatch repair-deficient crypt foci in lynch syndrome–molecular alterations and association with clinical parameters. PloS One (2015) 10(3):e0121980. doi: 10.1371/journal.pone.0121980 25816162 PMC4376900

[47] Ahadova A von Knebel Doeberitz M Bläker H Kloor M. CTNNB1-mutant colorectal carcinomas with immediate invasive growth: a model of interval cancers in lynch syndrome. Fam Cancer. (2016) 15(4):579–86. doi: 10.1007/s10689-016-9899-z 26960970

[48] Ten Broeke SW van Bavel TC Jansen AML Gómez-García E Hes FJ van Hest LP. Molecular background of colorectal tumors from patients with lynch syndrome associated with germline variants in PMS2. Gastroenterology (2018) 155(3):844–51. doi: 10.1053/j.gastro.2018.05.020 29758216

[49] Alexandrov LB Nik-Zainal S Wedge DC Aparicio SA Behjati S Biankin AV. Signatures of mutational processes in human cancer. Nature (2013) 500(7463):415–21. doi: 10.1038/nature12477 PMC3776390 23945592

[50] Bajwa-Ten Broeke SW Ballhausen A Ahadova A Suerink M Bohaumilitzky L Seidler F. The coding microsatellite mutation profile of PMS2-deficient colorectal cancer. Exp Mol Pathol (2021) 122:104668. doi: 10.1016/j.yexmp.2021.104668 34302852

[51] Alpert L Pai RK Srivastava A McKinnon W Wilcox R Yantiss RK. Colorectal carcinomas with isolated loss of PMS2 staining by immunohistochemistry. Arch Pathol Lab Med (2018) 142(4):523–8. doi: 10.5858/arpa.2017-0156-OA 29336605

[52] Wong S Hui P Buza N. Frequent loss of mutation-specific mismatch repair protein expression in nonneoplastic endometrium of lynch syndrome patients. Mod Pathol (2020) 33(6):1172–81. doi: 10.1038/s41379-020-0455-x 31932681

[53] Crosbie EJ Ryan NAJ Arends MJ Bosse T Burn J Cornes JM. The manchester international consensus group recommendations for the management of gynecological cancers in lynch syndrome. Genet Med (2019) 21(10):2390–400. doi: 10.1038/s41436-019-0489-y PMC6774998 30918358

[54] van Leerdam ME Roos VH van Hooft JE Balaguer F Dekker E Kaminski MF. Endoscopic management of lynch syndrome and of familial risk of colorectal cancer: European society of gastrointestinal endoscopy (ESGE) guideline. Endoscopy (2019) 51(11):1082–93. doi: 10.1055/a-1016-4977 31597170

[55] Monahan KJ Bradshaw N Dolwani S Desouza B Dunlop MG East JE. Guidelines for the management of hereditary colorectal cancer from the british society of gastroenterology (BSG)/Association of coloproctology of great britain and ireland (ACPGBI)/United kingdom cancer genetics group (UKCGG). Gut (2020) 69(3):411–44. doi: 10.1136/gutjnl-2019-319915 PMC7034349 31780574

[56] Seppälä TT Latchford A Negoi I Sampaio Soares A Jimenez-Rodriguez R Sánchez-Guillén L. European guidelines from the EHTG and ESCP for lynch syndrome: an updated third edition of the mallorca guidelines based on gene and gender. Br J Surg (2021) 108(5):484–98. doi: 10.1002/bjs.11902 PMC10364896 34043773

[57] Kang YJ Caruana M McLoughlin K Killen J Simms K Taylor N. The predicted effect and cost-effectiveness of tailoring colonoscopic surveillance according to mismatch repair gene in patients with lynch syndrome. Genet Med (2022) 24(9):1831–46. doi: 10.1016/j.gim.2022.05.016 35809086

[58] Kastrinos F Ingram MA Silver ER Oh A Laszkowska M Rustgi AK. Gene-specific variation in colorectal cancer surveillance strategies for lynch syndrome. Gastroenterology (2021) 161(2):453–462.e15. doi: 10.1053/j.gastro.2021.04.010 33839100 PMC9330543

[59] Miller DT Lee K Abul-Husn NS Amendola LM Brothers K Chung WK. ACMG SF v3.1 list for reporting of secondary findings in clinical exome and genome sequencing: A policy statement of the american college of medical genetics and genomics (ACMG). Genet Med (2022) 24(7):1407–14. doi: 10.1016/j.gim.2022.04.006 35802134

[60] Heneghan HM Martin ST Winter DC. Segmental vs extended colectomy in the management of hereditary nonpolyposis colorectal cancer: a systematic review and meta-analysis. Colorectal Dis (2015) 17(5):382–9. doi: 10.1111/codi.12868 25510173

[61] Anele CC Adegbola SO Askari A Rajendran A Clark SK Latchford A. Risk of metachronous colorectal cancer following colectomy in lynch syndrome: a systematic review and meta-analysis. Colorectal Dis (2017) 19(6):528–36. doi: 10.1111/codi.13679 28407411

[62] Malik SS Lythgoe MP McPhail M Monahan KJ. Metachronous colorectal cancer following segmental or extended colectomy in lynch syndrome: a systematic review and meta-analysis. Fam Cancer. (2018) 17(4):557–64. doi: 10.1007/s10689-017-0062-2 PMC6182577 29189962

[63] Te Paske IBAW Mensenkamp AR Neveling K ERN-GENTURIS Lynch-Like Working Group Hoogerbrugge N Ligtenberg MJL. Noncoding aberrations in mismatch repair genes underlie a substantial part of the missing heritability in lynch syndrome. Gastroenterology (2022) 163(6):1691–1694.e7. doi: 10.1053/j.gastro.2022.08.041 36037994

[64] Kono N Arakawa K. Nanopore sequencing: Review of potential applications in functional genomics. Dev Growth Differ (2019) 61(5):316–26. doi: 10.1111/dgd.12608 31037722

[65] Jain M Koren S Miga KH Quick J Rand AC Sasani TA. Nanopore sequencing and assembly of a human genome with ultra-long reads. Nat Biotechnol (2018) 36(4):338–45. doi: 10.1038/nbt.4060 PMC5889714 29431738

[66] Leija-Salazar M Sedlazeck FJ Toffoli M Mullin S Mokretar K Athanasopoulou M. Evaluation of the detection of GBA missense mutations and other variants using the oxford nanopore MinION. Mol Genet Genomic Med (2019) 7(3):e564. doi: 10.1002/mgg3.564 30637984 PMC6418358

[67] Perrod G Samaha E Rahmi G Khater S Abbes L Savale C. Impact of an optimized colonoscopic screening program for patients with lynch syndrome: 6-year results of a specialized french network. Therap Adv Gastroenterol (2018) 11:1756284818775058. doi: 10.1177/1756284818775058 PMC5974573 29872454

[68] Rivero-Sánchez L Arnau-Collell C Herrero J Remedios D Cubiella J García-Cougil M. White-light endoscopy is adequate for lynch syndrome surveillance in a randomized and noninferiority study. Gastroenterology (2020) 158(4):895–904.e1. doi: 10.1053/j.gastro.2019.09.003 31520613

[69] Burn J Mathers J Bishop DT. Lynch syndrome: history, causes, diagnosis, treatment and prevention (CAPP2 trial). Dig Dis (2012) 30 Suppl 2:39–47. doi: 10.1159/000341892 23207931

[70] Burn J Sheth H Elliott F Reed L Macrae F Mecklin JP. Cancer prevention with aspirin in hereditary colorectal cancer (Lynch syndrome), 10-year follow-up and registry-based 20-year data in the CAPP2 study: a double-blind, randomised, placebo-controlled trial. Lancet (2020) 395(10240):1855–63. doi: 10.1016/S0140-6736(20)30366-4 PMC7294238 32534647

[71] Schwitalle Y Kloor M Eiermann S Linnebacher M Kienle P Knaebel HP. Immune response against frameshift-induced neopeptides in HNPCC patients and healthy HNPCC mutation carriers. Gastroenterology (2008) 134(4):988–97. doi: 10.1053/j.gastro.2008.01.015 18395080

[72] Kloor M Reuschenbach M Pauligk C Karbach J Rafiyan MR Al-Batran SE. A frameshift peptide neoantigen-based vaccine for mismatch repair-deficient cancers: A phase I/IIa clinical trial. Clin Cancer Res (2020) 26(17):4503–10. doi: 10.1158/1078-0432.CCR-19-3517 32540851

[73] Gebert J Gelincik O Oezcan-Wahlbrink M Marshall JD Hernandez-Sanchez A Urban K. Recurrent frameshift neoantigen vaccine elicits protective immunity with reduced tumor burden and improved overall survival in a lynch syndrome mouse model. Gastroenterology (2021) 161(4):1288–1302.e13. doi: 10.1053/j.gastro.2021.06.073 34224739 PMC10184299

[74] Dudley JC Lin MT Le DT Eshleman JR. Microsatellite instability as a biomarker for PD-1 blockade. Clin Cancer Res (2016) 22(4):813–20. doi: 10.1158/1078-0432.CCR-15-1678 26880610

[75] Gelsomino F Barbolini M Spallanzani A Pugliese G Cascinu S. The evolving role of microsatellite instability in colorectal cancer: A review. Cancer Treat Rev (2016) 51:19–26. doi: 10.1016/j.ctrv.2016.10.005 27838401

[76] Liu X He S Wu H Xie H Zhang T Deng Z. Blocking the PD-1/PD-L1 axis enhanced cisplatin chemotherapy in osteosarcoma in vitro and *in vivo* . Environ Health Prev Med (2019) 24(1):79. doi: 10.1186/s12199-019-0835-3 31864288 PMC6925464

[77] Le DT Kim TW Van Cutsem E Geva R Jäger D Hara H. Phase II open-label study of pembrolizumab in treatment-refractory, microsatellite instability-High/Mismatch repair-deficient metastatic colorectal cancer: KEYNOTE-164. J Clin Oncol (2020) 38(1):11–9. doi: 10.1200/JCO.19.02107 PMC7031958 31725351

[78] Marabelle A Le DT Ascierto PA Di Giacomo AM De Jesus-Acosta A Delord JP. Efficacy of pembrolizumab in patients with noncolorectal high microsatellite Instability/Mismatch repair-deficient cancer: Results from the phase II KEYNOTE-158 study. J Clin Oncol (2020) 38(1):1–10. doi: 10.1200/JCO.19.02105 31682550 PMC8184060

[79] O'Malley DM Bariani GM Cassier PA Marabelle A Hansen AR De Jesus Acosta A. Pembrolizumab in patients with microsatellite instability-high advanced endometrial cancer: Results from the KEYNOTE-158 study. J Clin Oncol (2022) 40(7):752–61. doi: 10.1200/JCO.21.01874 PMC8887941 34990208

[80] Gonzalez A Kapila N Melendez-Rosado J Liang H Castro-Pavia F. An evaluation of the fecal microbiome in lynch syndrome. J Gastrointest Cancer. (2021) 52(1):365–8. doi: 10.1007/s12029-021-00588-z 33492618

[81] Yan Y Drew DA Markowitz A Lloyd-Price J Abu-Ali G Nguyen LH. Structure of the mucosal and stool microbiome in lynch syndrome. Cell Host Microbe (2020) 27(4):585–600.e4. doi: 10.1016/j.chom.2020.03.005 32240601 PMC7453618

[82] van Duijnhoven FJ Botma A Winkels R Nagengast FM Vasen HF Kampman E. Do lifestyle factors influence colorectal cancer risk in lynch syndrome? Fam Cancer (2013) 12(2):285–93. doi: 10.1007/s10689-013-9645-8 23657759

[83] Dashti SG Win AK Hardikar SS Glombicki SE Mallenahalli S Thirumurthi S. Physical activity and the risk of colorectal cancer in lynch syndrome. Int J Cancer. (2018) 143(9):2250–60. doi: 10.1002/ijc.31611 PMC6195467 29904935

[84] Day SD Enos RT McClellan JL Steiner JL Velázquez KT Murphy EA. Linking inflammation to tumorigenesis in a mouse model of high-fat-diet-enhanced colon cancer. Cytokine (2013) 64(1):454–62. doi: 10.1016/j.cyto.2013.04.031 PMC4826024 23735174

[85] Brouwer JG Makama M van Woudenbergh GJ Vasen HF Nagengast FM Kleibeuker JH. Inflammatory potential of the diet and colorectal tumor risk in persons with lynch syndrome. Am J Clin Nutr (2017) 106(5):1287–94. doi: 10.3945/ajcn.117.152900 28931533

[86] Botma A Vasen HF van Duijnhoven FJ Kleibeuker JH Nagengast FM Kampman E. Dietary patterns and colorectal adenomas in lynch syndrome: the GEOLynch cohort study. Cancer (2013) 119(3):512–21. doi: 10.1002/cncr.27726 23254892

[87] Mathers JC Elliott F Macrae F Mecklin JP Möslein G McRonald FE. Cancer prevention with resistant starch in lynch syndrome patients in the CAPP2-randomized placebo controlled trial: Planned 10-year follow-up. Cancer Prev Res (Phila). (2022) 15(9):623–34. doi: 10.1158/1940-6207.CAPR-22-0044 PMC9433960 35878732

[88] Mathers JC Movahedi M Macrae F Mecklin JP Moeslein G Olschwang S. Long-term effect of resistant starch on cancer risk in carriers of hereditary colorectal cancer: an analysis from the CAPP2 randomised controlled trial. Lancet Oncol (2012) 13(12):1242–9. doi: 10.1016/S1470-2045(12)70475-8 23140761

[89] Bounous VE Robba E Perotto S Pasini B Tomasi Cont N Ricci MT. Gynecological cancers in lynch syndrome: A comparison of the histological features with sporadic cases of the general population. J Clin Med (2022) 11(13):3689. doi: 10.3390/jcm11133689 35806973 PMC9267402

[90] Dashti SG Chau R Ouakrim DA Buchanan DD Clendenning M Young JP. Female hormonal factors and the risk of endometrial cancer in lynch syndrome. JAMA (2015) 314(1):61–71. doi: 10.1001/jama.2015.6789 26151267 PMC4688894

[91] Deltas C. Digenic inheritance and genetic modifiers. Clin Genet (2018) 93(3):429–38. doi: 10.1111/cge.13150 28977688

[92] Vogelaar IP Greer S Wang F Shin G Lau B Hu Y. Large cancer pedigree involving multiple cancer genes including likely digenic *MSH2* and *MSH6* lynch syndrome (LS) and an instance of recombinational rescue from LS. Cancers (Basel). (2022) 15(1):228. doi: 10.3390/cancers15010228 36612224 PMC9818763

[93] Schamschula E Kinzel M Wernstedt A Oberhuber K Gottschling H Schnaiter S. Teenage-onset colorectal cancers in a digenic cancer predisposition syndrome provide clues for the interaction between mismatch repair and polymerase δ proofreading deficiency in tumorigenesis. Biomolecules (2022) 12(10):1350. doi: 10.3390/biom12101350 36291559 PMC9599501

[94] Ahadova A Witt J Haupt S Gallon R Hüneburg R Nattermann J. Is HLA type a possible cancer risk modifier in lynch syndrome? Int J Cancer (2022). doi: 10.1002/ijc.34312 36214792

